# 2-[6-(4-Chloro­phen­yl)imidazo[2,1-*b*][1,3]thia­zol-2-yl]-*N*′-[(*E*)-4-meth­oxy­benzyl­idene]acetohydrazide

**DOI:** 10.1107/S1600536810052359

**Published:** 2010-12-18

**Authors:** Mehmet Akkurt, Nuray Ulusoy Güzeldemirci, Berin Karaman, Orhan Büyükgüngör

**Affiliations:** a Department of Physics, Faculty of Sciences, Erciyes University, 38039 Kayseri, Turkey; b Department of Pharmaceutical Chemistry, Faculty of Pharmacy, Istanbul University, 34116 Istanbul, Turkey; c Department of Physics, Faculty of Arts and Sciences, Ondokuz Mayıs University, 55139 Samsun, Turkey

## Abstract

In the imidazo[2,1-*b*][1,3]thia­zole group of the title compound, C_21_H_17_ClN_4_O_2_S, the dihedral angle between the thia­zole and imidazole rings is 1.9 (2)°. The mean plane of this group makes dihedral angles of 5.5 (2) and 39.9 (2)° with the benzene rings of the chloro­phenyl and meth­oxy­phenyl groups, respectively. The dihedral angle between these two benzene rings is 34.4 (2)°. In the crystal, mol­ecules are connected to each other by inter­molecular N—H⋯O hydrogen bonds along the *b* axis, generating a *C*(4) chain. Weak C—H⋯π inter­actions also occur.

## Related literature

For the biological activity of imidazo[2,1-*b*][1,3]thia­zole derivatives, see: Andreani *et al.* (2005 ▶); Barradas *et al.* (2008 ▶); Hanson *et al.* (1991 ▶); Juspin *et al.* (2010 ▶); Shilcrat *et al.* (1991 ▶). For details of the synthesis, see: Gürsoy & Ulusoy Güzeldemirci (2007 ▶); Ulusoy Güzeldemirci & Küçükbasmacı (2010 ▶). For related structures, see: Akkurt *et al.* (2007 ▶, 2008 ▶). For bond-length data, see: Allen *et al.* (1987 ▶).
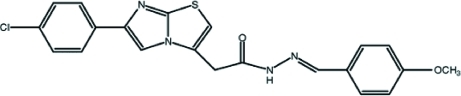


## Experimental

### 

#### Crystal data

C_21_H_17_ClN_4_O_2_S
*M* *_r_* = 424.91
Orthorhombic, 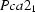

*a* = 13.4591 (6) Å
*b* = 4.7834 (3) Å
*c* = 30.4674 (14) Å
*V* = 1961.50 (18) Å^3^
*Z* = 4
Mo *K*α radiation
μ = 0.33 mm^−1^
*T* = 296 K
0.38 × 0.26 × 0.13 mm

#### Data collection

Stoe IPDS 2 diffractometer
Absorption correction: integration (*X-RED32*; Stoe & Cie, 2002 ▶) *T*_min_ = 0.890, *T*_max_ = 0.961
10765 measured reflections
3304 independent reflections
2754 reflections with *I* > 2σ(*I*)
*R*_int_ = 0.092

#### Refinement

*R*[*F* ^2^ > 2σ(*F* ^2^)] = 0.059
*wR*(*F* ^2^) = 0.164
*S* = 1.01
3304 reflections
265 parameters
1 restraint
H-atom parameters constrained
Δρ_max_ = 0.20 e Å^−3^
Δρ_min_ = −0.20 e Å^−3^
Absolute structure: Flack (1983 ▶), 1611 Friedel pairs
Flack parameter: 0.15 (11)

### 

Data collection: *X-AREA* (Stoe & Cie, 2002 ▶); cell refinement: *X-AREA*; data reduction: *X-RED32* (Stoe & Cie, 2002 ▶); program(s) used to solve structure: *SIR97* (Altomare *et al.*, 1999 ▶); program(s) used to refine structure: *SHELXL97* (Sheldrick, 2008 ▶); molecular graphics: *ORTEP-3* (Farrugia, 1997 ▶); software used to prepare material for publication: *WinGX* (Farrugia, 1999 ▶).

## Supplementary Material

Crystal structure: contains datablocks global, I. DOI: 10.1107/S1600536810052359/vm2066sup1.cif

Structure factors: contains datablocks I. DOI: 10.1107/S1600536810052359/vm2066Isup2.hkl

Additional supplementary materials:  crystallographic information; 3D view; checkCIF report

## Figures and Tables

**Table 1:** Hydrogen-bond geometry (Å, °) *Cg*1 is the centroid of the N1/N2/C7–C9 ring.

*D*—H⋯*A*	*D*—H	H⋯*A*	*D*⋯*A*	*D*—H⋯*A*
N3—H3*A*⋯O1^i^	0.86	2.08	2.835 (4)	146
C12—H12*A*⋯*Cg*1^i^	0.97	2.54	3.282 (5)	133

Symmetry code: (i) 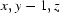.

**Fig. 1.**
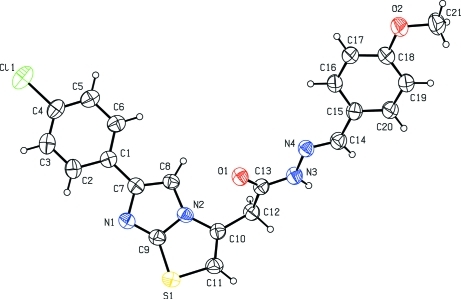
View of the title molecule, with the atom numbering scheme. Displacement ellipsoids for non-H atoms are drawn at the 30% probability level.

**Fig. 2.**
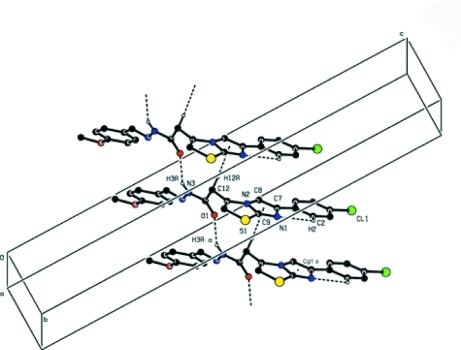
Partial view of the crystal packing showing the N—H···O, C—H···N hydrogen bonding interactions and C—H···π interactions of (I) in the unit cell. All H atoms not involved in hydrogen bonding are omitted for clarity. Cg1 is the centroid of ring N1/N2/C7—C9. [Symmetry code: (a) x, 1 + y, z].

## supplementary crystallographic information

### Comment

Imidazo[2,1-*b*][1,3]thiazole derivatives have demonstrated a broad range of biological activities, including immunomodulatory (Hanson *et al.*, 1991), antibacterial (Juspin *et al.*, 2010), antiviral (Barradas, *et al.*., 2008), antitumoral (Andreani *et al.*, 2005) and antiinflammatory (Shilcrat *et al.*, 1991).

As part of our on-going research aiming on the synthesis of imidazo[2,1-*b*]thiazoles (Gürsoy & Ulusoy Güzeldemirci, 2007; Ulusoy Güzeldemirci & Küçükbasmacı, 2010) and their crystal structures (Akkurt *et al.*, 2007, 2008), we report here the crystal structure of the title compound (I).

In the title molecule of (I), (Fig. 1), the bond lengths and bond angles are normal (Allen *et al.*, 1987). The imidazo[2,1-*b*][1,3]thiazole group is essantially planar with a dihedral angle of 1.9 (2) ° between the thiazole and imidazole rings makes a dihedral angle of 2.99 (15)°.

The mean plane of the imidazo[2,1-*b*][1,3]thiazole group makes dihedral angles of 5.5 (2) and 39.9 (2) ° with the benzene rings of the chlorophenyl and methoxyphenyl groups, respectively. The dihedral angle between these two phenyl rings is 34.4 (2) °.

Molecules are connected to each other by intermolecular N—H···O hydrogen bonds along the *b* axis, generating a C(4) chain (Table 1, Fig. 2). In the crystal structure, a weak C—H···π interaction occurs.

### Experimental

[6-(4-Chlorophenyl)imidazo[2,1-*b*]thiazol-3-yl]acetic acid hydrazide (0.005 mol) and 4-methoxybenzaldehyde (0.005 mol) were heated in 100 ml ethanol for 5 h. The precipitate obtained was purified by washing with hot ethanol. Yield: 88%. *M*.p.: 511.5–512.6 K. IR [ν, cm^-1^, KBr]: 3212, 3138 (N—H), 1660 (C=O). Analysis calculated for C_21_H_17_ClN_4_O_2_S: C 59.36, H 4.03, N 13.19%. Found: C 59.25, H 3.94, N 13.18%.

### Refinement

All H atoms were placed in geometrically idealized positions and refined using a riding model, with N—H = 0.86 Å and C—H = 0.93, 0.97 and 0.96 Å for aromatic, methylene and methyl H, respectively, and *U*_iso_(H) = 1.5*U*_eq_(C) for methyl H, and *U*_iso_(H) =1.2*U*_eq_(C,*N*) for all other H atoms.

### Crystal data

**Table:**

C_21_H_17_ClN_4_O_2_S	*F*(000) = 880
*M**_r_* = 424.91	*D*_x_ = 1.439 Mg m^−^^3^
Orthorhombic, *P**c**a*2_1_	Mo *K*α radiation, λ = 0.71073 Å
Hall symbol: P 2c -2ac	Cell parameters from 15357 reflections
*a* = 13.4591 (6) Å	θ = 1.3–25.1°
*b* = 4.7834 (3) Å	µ = 0.33 mm^−^^1^
*c* = 30.4674 (14) Å	*T* = 296 K
*V* = 1961.50 (18) Å^3^	Prism, colourless
*Z* = 4	0.38 × 0.26 × 0.13 mm

### Data collection

**Table:**

Stoe IPDS 2 diffractometer	3304 independent reflections
Radiation source: sealed X-ray tube, 12 x 0.4 mm long-fine focus	2754 reflections with *I* > 2σ(*I*)
plane graphite	*R*_int_ = 0.092
Detector resolution: 6.67 pixels mm^-1^	θ_max_ = 24.6°, θ_min_ = 1.3°
ω scans	*h* = −15→15
Absorption correction: integration (*X-RED32*; Stoe & Cie, 2002)	*k* = −5→5
*T*_min_ = 0.890, *T*_max_ = 0.961	*l* = −35→35
10765 measured reflections	

### Refinement

**Table:**

Refinement on *F*^2^	Hydrogen site location: inferred from neighbouring sites
Least-squares matrix: full	H-atom parameters constrained
*R*[*F*^2^ > 2σ(*F*^2^)] = 0.059	*w* = 1/[σ^2^(*F*_o_^2^) + (0.1136*P*)^2^] where *P* = (*F*_o_^2^ + 2*F*_c_^2^)/3
*wR*(*F*^2^) = 0.164	(Δ/σ)_max_ = 0.001
*S* = 1.01	Δρ_max_ = 0.20 e Å^−^^3^
3304 reflections	Δρ_min_ = −0.20 e Å^−^^3^
265 parameters	Extinction correction: *SHELXL97* (Sheldrick, 2008), FC^*^=KFC[1+0.001XFC^2^Λ^3^/SIN(2Θ)]^-1/4^
1 restraint	Extinction coefficient: 0.010 (2)
Primary atom site location: structure-invariant direct methods	Absolute structure: Flack (1983), 1611 Friedel pairs
Secondary atom site location: difference Fourier map	Flack parameter: 0.15 (11)

### Special details

**Table:**

Geometry. Bond distances, angles *etc*. have been calculated using the rounded fractional coordinates. All su's are estimated from the variances of the (full) variance-covariance matrix. The cell e.s.d.'s are taken into account in the estimation of distances, angles and torsion angles
Refinement. Refinement on *F*^2^ for ALL reflections except those flagged by the user for potential systematic errors. Weighted *R*-factors *wR* and all goodnesses of fit *S* are based on *F*^2^, conventional *R*-factors *R* are based on *F*, with *F* set to zero for negative *F*^2^. The observed criterion of *F*^2^ > σ(*F*^2^) is used only for calculating -*R*-factor-obs *etc*. and is not relevant to the choice of reflections for refinement. *R*-factors based on *F*^2^ are statistically about twice as large as those based on *F*, and *R*-factors based on ALL data will be even larger.

### Fractional atomic coordinates and isotropic or equivalent isotropic displacement parameters (Å^2^)

**Table:**

	*x*	*y*	*z*	*U*_iso_*/*U*_eq_	
Cl1	0.54087 (14)	1.5430 (3)	0.72891 (5)	0.1113 (6)	
S1	1.01901 (8)	0.7353 (3)	0.53219 (5)	0.0824 (4)	
O1	0.6911 (2)	0.6101 (5)	0.46534 (10)	0.0706 (10)	
O2	0.1795 (3)	0.1793 (9)	0.30456 (13)	0.0947 (12)	
N1	0.8812 (3)	0.9668 (8)	0.59159 (12)	0.0709 (11)	
N2	0.8350 (2)	0.6242 (7)	0.54579 (11)	0.0612 (10)	
N3	0.6651 (3)	0.1742 (7)	0.43926 (12)	0.0689 (11)	
N4	0.5793 (3)	0.2476 (7)	0.41650 (13)	0.0676 (11)	
C1	0.7215 (3)	1.0549 (9)	0.62971 (14)	0.0647 (12)	
C2	0.7662 (4)	1.2522 (10)	0.65723 (15)	0.0770 (14)	
C3	0.7105 (4)	1.3995 (11)	0.68731 (17)	0.0863 (19)	
C4	0.6104 (4)	1.3556 (10)	0.69031 (16)	0.0800 (19)	
C5	0.5640 (4)	1.1665 (12)	0.66394 (17)	0.0823 (17)	
C6	0.6196 (4)	1.0159 (11)	0.63367 (15)	0.0767 (17)	
C7	0.7810 (3)	0.9024 (9)	0.59711 (15)	0.0653 (12)	
C8	0.7513 (3)	0.6957 (8)	0.56975 (14)	0.0630 (12)	
C9	0.9110 (3)	0.7939 (9)	0.56040 (15)	0.0680 (12)	
C10	0.8635 (3)	0.4461 (8)	0.51164 (14)	0.0620 (12)	
C11	0.9596 (3)	0.4809 (10)	0.50111 (17)	0.0720 (17)	
C12	0.7937 (3)	0.2362 (8)	0.49197 (16)	0.0655 (14)	
C13	0.7124 (3)	0.3618 (8)	0.46392 (14)	0.0596 (11)	
C14	0.5417 (4)	0.0495 (10)	0.39367 (16)	0.0723 (16)	
C15	0.4478 (4)	0.0818 (9)	0.37059 (15)	0.0700 (14)	
C16	0.3780 (4)	0.2839 (9)	0.38208 (16)	0.0730 (16)	
C17	0.2884 (4)	0.3090 (11)	0.36042 (17)	0.0790 (17)	
C18	0.2698 (4)	0.1353 (10)	0.32490 (17)	0.0750 (16)	
C19	0.3377 (4)	−0.0638 (10)	0.31242 (17)	0.0817 (17)	
C20	0.4246 (4)	−0.0884 (10)	0.33545 (18)	0.0783 (17)	
C21	0.1516 (5)	−0.0120 (16)	0.2706 (2)	0.115 (3)	
H2	0.83420	1.28430	0.65520	0.0920*	
H3	0.74110	1.52900	0.70560	0.1030*	
H3A	0.68770	0.00650	0.43740	0.0830*	
H5	0.49580	1.13840	0.66620	0.0980*	
H6	0.58800	0.88640	0.61570	0.0920*	
H8	0.68810	0.61820	0.56750	0.0760*	
H11	0.99130	0.37860	0.47930	0.0860*	
H12A	0.76320	0.13000	0.51550	0.0780*	
H12B	0.83180	0.10670	0.47420	0.0780*	
H14	0.57540	−0.11980	0.39180	0.0870*	
H16	0.39220	0.40560	0.40500	0.0880*	
H17	0.24140	0.43960	0.36950	0.0950*	
H19	0.32490	−0.18020	0.28860	0.0980*	
H20	0.46990	−0.22540	0.32710	0.0940*	
H21A	0.20130	−0.01150	0.24800	0.1720*	
H21B	0.08890	0.04400	0.25840	0.1720*	
H21C	0.14580	−0.19670	0.28270	0.1720*	

### Atomic displacement parameters (Å^2^)

**Table:**

	*U*^11^	*U*^22^	*U*^33^	*U*^12^	*U*^13^	*U*^23^
Cl1	0.1479 (14)	0.0934 (9)	0.0926 (8)	0.0296 (9)	0.0312 (9)	−0.0020 (8)
S1	0.0569 (5)	0.0919 (8)	0.0983 (8)	−0.0039 (5)	0.0019 (6)	−0.0118 (7)
O1	0.0780 (18)	0.0475 (13)	0.0862 (18)	0.0048 (13)	−0.0115 (16)	0.0021 (14)
O2	0.086 (2)	0.102 (2)	0.096 (2)	0.004 (2)	−0.020 (2)	−0.020 (2)
N1	0.0618 (18)	0.076 (2)	0.075 (2)	0.0001 (17)	−0.0067 (18)	−0.005 (2)
N2	0.0554 (17)	0.0586 (16)	0.0697 (19)	−0.0003 (14)	−0.0055 (15)	0.0055 (15)
N3	0.075 (2)	0.0517 (17)	0.080 (2)	0.0084 (15)	−0.0098 (19)	0.0007 (16)
N4	0.069 (2)	0.0569 (17)	0.077 (2)	0.0017 (15)	−0.0060 (17)	−0.0023 (17)
C1	0.068 (2)	0.063 (2)	0.063 (2)	0.0025 (19)	−0.0017 (18)	0.0064 (18)
C2	0.093 (3)	0.068 (2)	0.070 (2)	−0.006 (2)	−0.004 (2)	0.000 (2)
C3	0.108 (4)	0.077 (3)	0.074 (3)	0.001 (3)	−0.007 (3)	−0.010 (3)
C4	0.099 (4)	0.070 (3)	0.071 (3)	0.017 (2)	0.009 (3)	0.002 (2)
C5	0.079 (3)	0.088 (3)	0.080 (3)	0.015 (3)	0.007 (3)	0.003 (3)
C6	0.074 (3)	0.083 (3)	0.073 (3)	0.006 (2)	0.005 (2)	0.003 (2)
C7	0.067 (2)	0.062 (2)	0.067 (2)	0.0017 (18)	−0.004 (2)	0.007 (2)
C8	0.057 (2)	0.062 (2)	0.070 (2)	−0.0038 (18)	0.0016 (18)	0.0042 (19)
C9	0.060 (2)	0.069 (2)	0.075 (2)	0.0014 (19)	−0.003 (2)	0.003 (2)
C10	0.066 (2)	0.055 (2)	0.065 (2)	0.0055 (17)	−0.0037 (19)	0.0025 (18)
C11	0.062 (3)	0.072 (3)	0.082 (3)	0.0067 (19)	−0.002 (2)	0.000 (2)
C12	0.068 (2)	0.0504 (19)	0.078 (3)	0.0026 (17)	−0.006 (2)	0.0034 (18)
C13	0.066 (2)	0.0478 (18)	0.065 (2)	−0.0003 (16)	0.0044 (19)	0.0037 (17)
C14	0.076 (3)	0.064 (2)	0.077 (3)	0.002 (2)	−0.001 (2)	−0.002 (2)
C15	0.078 (3)	0.061 (2)	0.071 (2)	−0.001 (2)	0.000 (2)	0.000 (2)
C16	0.077 (3)	0.068 (2)	0.074 (3)	−0.002 (2)	−0.005 (2)	−0.012 (2)
C17	0.072 (3)	0.076 (3)	0.089 (3)	0.005 (2)	−0.003 (2)	−0.013 (2)
C18	0.071 (3)	0.080 (3)	0.074 (2)	−0.006 (2)	−0.010 (2)	−0.001 (2)
C19	0.080 (3)	0.081 (3)	0.084 (3)	0.000 (2)	−0.009 (2)	−0.018 (2)
C20	0.074 (3)	0.068 (3)	0.093 (3)	0.005 (2)	−0.005 (2)	−0.013 (2)
C21	0.113 (5)	0.123 (5)	0.108 (4)	0.001 (4)	−0.034 (4)	−0.034 (4)

### Geometric parameters (Å, °)

**Table:**

Cl1—C4	1.750 (5)	C12—C13	1.513 (6)
S1—C9	1.712 (4)	C14—C15	1.455 (7)
S1—C11	1.737 (5)	C15—C20	1.381 (7)
O1—C13	1.223 (5)	C15—C16	1.393 (7)
O2—C18	1.380 (7)	C16—C17	1.380 (7)
O2—C21	1.431 (8)	C17—C18	1.387 (7)
N1—C7	1.394 (6)	C18—C19	1.374 (7)
N1—C9	1.322 (6)	C19—C20	1.369 (8)
N2—C8	1.385 (5)	C2—H2	0.9300
N2—C9	1.380 (5)	C3—H3	0.9300
N2—C10	1.398 (5)	C5—H5	0.9300
N3—N4	1.392 (6)	C6—H6	0.9300
N3—C13	1.332 (5)	C8—H8	0.9300
N4—C14	1.280 (6)	C11—H11	0.9300
N3—H3A	0.8600	C12—H12A	0.9700
C1—C6	1.389 (7)	C12—H12B	0.9700
C1—C7	1.470 (6)	C14—H14	0.9300
C1—C2	1.399 (6)	C16—H16	0.9300
C2—C3	1.378 (7)	C17—H17	0.9300
C3—C4	1.367 (8)	C19—H19	0.9300
C4—C5	1.362 (7)	C20—H20	0.9300
C5—C6	1.389 (7)	C21—H21A	0.9600
C7—C8	1.354 (6)	C21—H21B	0.9600
C10—C12	1.500 (6)	C21—H21C	0.9600
C10—C11	1.343 (6)		
			
C9—S1—C11	89.9 (2)	C16—C17—C18	118.6 (5)
C18—O2—C21	117.3 (5)	O2—C18—C19	124.6 (5)
C7—N1—C9	104.0 (4)	O2—C18—C17	114.7 (5)
C8—N2—C9	106.7 (3)	C17—C18—C19	120.7 (5)
C8—N2—C10	140.0 (3)	C18—C19—C20	119.1 (5)
C9—N2—C10	113.3 (3)	C15—C20—C19	122.7 (5)
N4—N3—C13	120.5 (3)	C1—C2—H2	120.00
N3—N4—C14	114.3 (4)	C3—C2—H2	120.00
C13—N3—H3A	120.00	C2—C3—H3	120.00
N4—N3—H3A	120.00	C4—C3—H3	120.00
C2—C1—C7	120.4 (4)	C4—C5—H5	120.00
C2—C1—C6	117.6 (4)	C6—C5—H5	120.00
C6—C1—C7	122.0 (4)	C1—C6—H6	119.00
C1—C2—C3	120.7 (5)	C5—C6—H6	119.00
C2—C3—C4	120.2 (5)	N2—C8—H8	127.00
C3—C4—C5	121.0 (5)	C7—C8—H8	127.00
Cl1—C4—C5	119.5 (4)	S1—C11—H11	123.00
Cl1—C4—C3	119.6 (4)	C10—C11—H11	123.00
C4—C5—C6	119.3 (5)	C10—C12—H12A	109.00
C1—C6—C5	121.3 (5)	C10—C12—H12B	109.00
N1—C7—C1	120.0 (4)	C13—C12—H12A	109.00
N1—C7—C8	111.9 (4)	C13—C12—H12B	109.00
C1—C7—C8	128.2 (4)	H12A—C12—H12B	108.00
N2—C8—C7	105.4 (3)	N4—C14—H14	119.00
N1—C9—N2	112.0 (4)	C15—C14—H14	119.00
S1—C9—N1	136.1 (3)	C15—C16—H16	119.00
S1—C9—N2	111.8 (3)	C17—C16—H16	119.00
N2—C10—C11	111.5 (4)	C16—C17—H17	121.00
N2—C10—C12	122.2 (3)	C18—C17—H17	121.00
C11—C10—C12	126.2 (4)	C18—C19—H19	120.00
S1—C11—C10	113.6 (4)	C20—C19—H19	120.00
C10—C12—C13	114.4 (3)	C15—C20—H20	119.00
N3—C13—C12	113.4 (3)	C19—C20—H20	119.00
O1—C13—C12	122.4 (4)	O2—C21—H21A	110.00
O1—C13—N3	124.2 (4)	O2—C21—H21B	109.00
N4—C14—C15	121.9 (4)	O2—C21—H21C	109.00
C16—C15—C20	116.9 (5)	H21A—C21—H21B	110.00
C14—C15—C16	122.6 (4)	H21A—C21—H21C	110.00
C14—C15—C20	120.6 (4)	H21B—C21—H21C	109.00
C15—C16—C17	122.0 (4)		
			
C11—S1—C9—N2	−0.2 (3)	C6—C1—C7—C8	−5.7 (7)
C11—S1—C9—N1	−177.4 (5)	C2—C1—C7—C8	176.1 (4)
C9—S1—C11—C10	0.4 (4)	C1—C2—C3—C4	−0.5 (7)
C21—O2—C18—C17	−173.8 (5)	C2—C3—C4—Cl1	179.6 (4)
C21—O2—C18—C19	5.6 (8)	C2—C3—C4—C5	0.2 (8)
C7—N1—C9—N2	0.2 (5)	Cl1—C4—C5—C6	−179.3 (4)
C7—N1—C9—S1	177.4 (4)	C3—C4—C5—C6	0.2 (8)
C9—N1—C7—C1	−180.0 (4)	C4—C5—C6—C1	−0.2 (8)
C9—N1—C7—C8	−0.1 (5)	C1—C7—C8—N2	179.9 (4)
C9—N2—C8—C7	0.1 (4)	N1—C7—C8—N2	0.0 (5)
C10—N2—C9—N1	177.8 (4)	C11—C10—C12—C13	−111.4 (5)
C10—N2—C8—C7	−177.0 (5)	N2—C10—C11—S1	−0.6 (5)
C8—N2—C9—S1	−178.1 (3)	C12—C10—C11—S1	−177.6 (3)
C9—N2—C10—C12	177.6 (4)	N2—C10—C12—C13	71.8 (5)
C10—N2—C9—S1	−0.1 (4)	C10—C12—C13—O1	−16.2 (6)
C8—N2—C9—N1	−0.2 (5)	C10—C12—C13—N3	166.1 (4)
C8—N2—C10—C12	−5.4 (7)	N4—C14—C15—C20	158.2 (5)
C9—N2—C10—C11	0.4 (5)	N4—C14—C15—C16	−21.1 (7)
C8—N2—C10—C11	177.4 (5)	C14—C15—C20—C19	−179.3 (5)
N4—N3—C13—O1	−7.0 (7)	C16—C15—C20—C19	0.1 (7)
C13—N3—N4—C14	179.3 (4)	C14—C15—C16—C17	−178.8 (5)
N4—N3—C13—C12	170.6 (4)	C20—C15—C16—C17	1.9 (7)
N3—N4—C14—C15	175.0 (4)	C15—C16—C17—C18	−3.0 (8)
C2—C1—C7—N1	−4.1 (6)	C16—C17—C18—C19	2.1 (8)
C2—C1—C6—C5	−0.2 (7)	C16—C17—C18—O2	−178.5 (4)
C6—C1—C2—C3	0.5 (7)	O2—C18—C19—C20	−179.6 (5)
C7—C1—C6—C5	−178.4 (5)	C17—C18—C19—C20	−0.2 (8)
C7—C1—C2—C3	178.8 (4)	C18—C19—C20—C15	−0.9 (8)
C6—C1—C7—N1	174.1 (4)		

### Hydrogen-bond geometry (Å, °)

**Table:**

Cg1 is the centroid of the N1/N2/C7–C9 ring.

**Table:**

*D*—H···*A*	*D*—H	H···*A*	*D*···*A*	*D*—H···*A*
N3—H3A···O1^i^	0.86	2.08	2.835 (4)	146
C2—H2···N1	0.93	2.54	2.874 (6)	101
C12—H12A···Cg1^i^	0.97	2.54	3.282 (5)	133

Symmetry codes: (i) *x*, *y*−1, *z*.
